# Asymmetric growth-limiting development of the female conceptus

**DOI:** 10.3389/fendo.2023.1306513

**Published:** 2024-02-01

**Authors:** Consuelo Amor S. Estrella, Kathryn L. Gatford, Ruidong Xiang, Ali Javadmanesh, Mani Ghanipoor-Samami, Greg S. Nattrass, Entesar Shuaib, Milton M. McAllister, Ian Beckman, Dana A. Thomsen, Vicki L. Clifton, Julie A. Owens, Claire T. Roberts, Stefan Hiendleder, Karen L. Kind

**Affiliations:** ^1^ Robinson Research Institute, The University of Adelaide, Adelaide, SA, Australia; ^2^ Epigenetics and Genetics Group and Davies Research Centre, School of Animal and Veterinary Sciences, The University of Adelaide, Roseworthy, SA, Australia; ^3^ School of Biomedicine, The University of Adelaide, Adelaide, SA, Australia; ^4^ South Australian Research and Development Institute, Livestock Systems, Roseworthy, SA, Australia; ^5^ School of Animal and Veterinary Sciences, The University of Adelaide, Roseworthy, SA, Australia; ^6^ Mater Research Institute, University of Queensland, Brisbane, QLD, Australia; ^7^ Deakin University, Geelong, VIC, Australia; ^8^ Flinders University, College of Medicine and Public Health, Adelaide, SA, Australia

**Keywords:** conceptus, uncomplicated pregnancy, sex differences, asymmetric growth, IGF system, histomorphology, clinico-chemical screen

## Abstract

**Introduction:**

Sex differences in prenatal growth may contribute to sex-dependent programming effects on postnatal phenotype.

**Methods:**

We integrated for the first time phenotypic, histomorphological, clinico-chemical, endocrine and gene expression analyses in a single species, the bovine conceptus at mid-gestation.

**Results:**

We demonstrate that by mid-gestation, before the onset of accelerated growth, the female conceptus displays asymmetric lower growth compared to males. Female fetuses were smaller with lower ponderal index and organ weights than males. However, their brain:body weight, brain:liver weight and heart:body weight ratios were higher than in males, indicating brain and heart ‘sparing’. The female placenta weighed less and had lower volumes of trophoblast and fetal connective tissue than the male placenta. Female umbilical cord vessel diameters were smaller, and female-specific relationships of body weight and brain:liver weight ratios with cord vessel diameters indicated that the umbilico-placental vascular system creates a growth-limiting environment where blood flow is redistributed to protect brain and heart growth. Clinico-chemical indicators of liver perfusion support this female-specific growth-limiting phenotype, while lower insulin-like growth factor 2 (IGF2) gene expression in brain and heart, and lower circulating IGF2, implicate female-specific modulation of key endocrine mediators by nutrient supply.

**Conclusion:**

This mode of female development may increase resilience to environmental perturbations *in utero* and contribute to sex-bias in programming outcomes including susceptibility to non-communicable diseases.

## 1 Introduction

Epidemiological and experimental data obtained in humans and other species show that prenatal developmental plasticity and “programming” allow the development of different phenotypes from a given genotype, in response to environmental cues. The adaptive responses of a conceptus may manifest as postnatal physiological and morphological changes that are within the normal, healthy range of variation, or beyond, into categories of non-communicable disease such as insulin resistance and type 2 diabetes (1–4). Importantly, developmental programming effects on postnatal phenotype are frequently sex specific (5–9).

In humans, increased size of male fetuses is evident throughout gestation from 8 to 12 weeks to term (10, 11), with male fetuses being heavier, longer, and leaner than female fetuses at birth (10, 11). Sex differences in growth patterns for fetal biometric indices, including head and abdominal circumference, are reported from mid-gestation (10, 12, 13) and result in sex-specific percentiles for weight, length, and head circumference at birth (14).

The critical role of the placenta in delivering substrates for growth and development manifests in tight correlations between placental and fetal weights (15, 16). At birth, female fetuses have a lower placental weight and placental efficiency (15, 17–19), a shorter umbilical cord (20), and smaller cord vein diameter with a higher cord artery pulsatility index than male fetuses (21). Human epidemiological and experimental animal studies suggest that a slower growth pattern allows the female fetus to more readily adapt to perturbations of the *in utero* environment, compared with the male fetus (22), and it has been hypothesized that the placenta plays a key role in mediating sex-specific adaptation of fetal growth in response to environmental factors (23). The insulin-like growth factor (IGF) system is a major regulator of conceptus growth, with a dominant role of IGF2 in early fetal growth and development (24). Substrate supply modulation of the IGF system and, in turn, IGF regulation of fetal and placental growth are evident in a range of mammalian species and may contribute to sex-specific differences in growth and development (24).

Previously reported prenatal sex differences in fetal–placental phenotype of healthy pregnancies relied largely on data from rodents (25–27) or ultrasound-derived data in humans (10–13). Sex-specific integrated analyses of histomorphological, clinico-chemical, circulating IGF, and *IGF* tissue expression data in combination with extensive fetal-placental phenotypic data obtained in a single species are non-existent. Here, we performed these analyses in a large set of healthy mid-gestation concepti, with a broad range of fetal weights, using the outbred bovine, where dams carry a single fetus with gestation length, fetal growth curve, and maturity at birth similar to human (28–32), to better understand the basis and origin of sex differences in prenatal development that may contribute to sex bias in programming outcomes.

## 2 Materials and methods

### 2.1 Ethics statement

All animals and procedures used were approved by The University of Adelaide Animal Ethics Committee (No. S-094-2005).

### 2.2 Concepti

We used *Bos taurus taurus* (Angus, A) and *Bos taurus indicus* (Brahman, B) genetics to generate purebred and reciprocal cross concepti for comparison of placental and fetal parameters across a range of healthy fetal weights (**Supplementary Table S1**). Nulliparous dams at 16–20 months of age were purchased in South Australia and New South Wales and transferred to Struan Research Centre, Naracoorte, South Australia. Dams were managed in one group and grazed on pasture supplemented by hay silage. Pregnancies were established using three Angus and two Brahman sires after standard estrous cycle synchronization procedures following an adjustment period of 3–4 weeks from animal purchase (32). All pregnancies were confirmed by ultrasound, but sex was unknown until fetuses were recovered. We excluded two twin pregnancies and one pregnancy where the dam displayed unexplained weight loss. The experimental concepti collected at day 153 ± 1 post-insemination (term 279–291 days) (28) consisted of a total of 45 female and 27 male healthy singletons from uncomplicated pregnancies.

### 2.3 Collection of samples and phenotype data

Pregnant animals were fasted 24 h before humane killing under standardized conditions in an abattoir. Entire uteri were removed and opened by longitudinal incision and the fetus removed after clamping and cutting the umbilical cord immediately above the placenta. Cord blood samples were collected in Lithium-Heparin-LH and Serum Z S-Monovettes^®^ (Sarstedt, Nümbrecht, Germany) after cutting the cord above the clamp. Serum and plasma samples were obtained after centrifugation and stored frozen at −80°C until further analysis. Fetal weight, cord weight and length, and fetal organ weights (brain, heart, lungs, liver, and kidneys) were recorded, and organ samples, including skeletal muscle (*M. semitendinosus*) and placenta (cotyledon), were stored in RNAlater (Ambion, Thermo Fisher Scientific, Melbourne, Australia). The largest placentome close to the fetus was removed for histology and placed in a Petri dish with the chorionic plate facing up. A 5-mm-thick cross-section through the center of this placental sample was excised and fixed in ice-cold 4% paraformaldehyde and 2.5% PVP-40 PBS for 24 h. The sample was then washed four times with 1% PBS and stored in 70% ethanol until embedding in paraffin. The entire umbilical cord was washed in 1% PBS and stored in 70% ethanol until further analysis of cord vessels. After collecting and weighing fetal fluids, eviscerated fetuses and uteri with attached placentae were vacuum-packed and stored frozen at −22°C until dissection for muscle, bone, and placental parameters (see below). Ratios of organ to body weight were calculated by dividing organ weights by fetal weight. Brain to liver weight ratio was obtained in the same manner. Placental efficiency was calculated by dividing fetal weight by placental weight.

### 2.4 Fetal muscle, bone, and ponderal index

Four representative muscles/muscle groups from the front limb (*Musculus supraspinatus*), back (*M. longissimus dorsi*), and hind limb (*M. semimembranosus* and *M. quadriceps femoris*) were dissected and weighed (33). Fetal muscle mass was calculated as the sum of the weights of these muscles. A total of 12 bones, *Os mandibulare*, *Os scapulare*, *Os humeri*, *Os radiale*, *Os ulnare*, *Ossa metacarpalia*, *Os costale VI*, *Os pelvis*, *Os femoris*, *Os tibiale*, *Ossa metatarsalia*, and *Columna vertebralis* were removed and cleaned to obtain bone weights as described (32). Fetal bone mass was calculated as the sum of the weights of the 12 bones. Femur length was measured between the most distal points of epiphyses, and fetus length was measured as the length of the spinal column. Ponderal index (PI) was calculated as fetus weight, kg/(fetal length, m)^3^.

### 2.5 Umbilical cord and umbilical vessel diameters

Umbilical cord diameter was calculated from fresh cord length and weight using the formula previously reported (34). To obtain artery and vein diameters, umbilical cords were fixed in 70% ethanol and cross-sectioned at mid-length. The bovine umbilical vessels, two arteries and two veins, were identified, and the diameter of each cord vessel was measured using a caliper as described previously for sonographic assessment of human cord vessels (35, 36). Total artery and vein diameter are reported.

### 2.6 Immunohistochemistry and histomorphometric analysis of the placenta

Histomorphology of the placenta was assessed using indirect double immunohistochemistry on 5-µm-thick longitudinal slices of placentomes as described previously (16). Briefly, 10% porcine serum and 1% BSA in PBS served as a diluent and block to ensure non-specific binding. The primary antibodies used were the mouse anti-human Vimentin clone Vim3B4 (DakoCytomation, Glostrup, Denmark) and the mouse anti-human Cytokeratin AE1/AE3 (Millipore, Temecula, LA, USA) in 1:10 and 1:400 dilutions, respectively. The biotinylated goat anti-mouse (DakoCytomation, Glostrup, Denmark), in 1:200 dilution, was used as the secondary antibody. The placentomes were counterstained with hematoxylin (Sigma, St. Louis, MO, USA) and Eosin (Sigma, St. Louis, MO, USA). A total of 10 fields at ×200 magnification derived from a high-resolution image of the whole stained placentome section (NanoZoomer C9600 slide scanner, Hamamatsu Photonics K.K., Hamamatsu City, Japan) were analyzed for histomorphometric parameters including volume densities and volume of the different placental cell types, barrier thickness, and surface densities and surface areas. Each field was counted five times, and the coefficient of variation was < 5%.

### 2.7 Clinico-chemical parameters in the cord serum

Cord serum samples were assayed for electrolytes (total Ca, Cl, Mg, Na, P, and K), metabolites (albumin, cholesterol, creatinine, globulin, glucose, total protein, triglycerides, and urea), and enzymes (ALP, ALT, GGT, and GLDH) using the Beckman-Coulter AU Clinical Chemistry Analyzer AU 480 (Beckman Coulter, Lane Cove, Australia). Lactate and chloride were measured using a Radiometer 725 (Diamond Diagnostics, Holliston, MA, USA).

### 2.8 Insulin-like growth factors and total IGF-binding protein in the cord plasma

Concentrations of cord plasma IGF1, IGF2, and total IGF-binding protein (IGFBP) binding were measured by RIA after separation of IGFs and IGFBPs by size-exclusion HPLC under acidic conditions as described previously for neonatal bovine plasma samples (37). Recovery of ^125^I-IGF1 was 92.5% ± 0.5% for nine HPLC runs of fetal plasma. Samples were assayed in triplicate in each assay. Plasma IGF1 concentrations were measured by analysis of neutralized HPLC fraction 3, in a RIA specific for IGF1 using a rabbit polyclonal antibody to human IGF1 (GroPep, Adelaide, Australia) (38). Total IGFBP binding protein (tIGFBP) concentrations were measured by analysis of neutralized HPLC fraction 1 in the same assay. As IGFBPs bind to and sequester ^125^I-IGF1 in this assay, they can be measured due to their effect of reducing the amount of ^125^I-IGF1 in the immunoprecipitated pellet, giving an apparent IGF concentration that reflects the total amount and binding affinity of IGFBP present in plasma (39). The inter-assay CV for HPLC separation and RIA of IGF1 was 5.4% (n = 5 assays), and the intra-assay CV for extraction and assay was 10.9% for a neonatal bovine plasma QC sample containing 43.9 ng/mL of IGF1. Plasma IGF2 concentrations were measured by analysis of HPLC fraction 3 in a RIA specific for IGF2 (40). The inter-assay CV for HPLC separation and RIA of IGF2 was 2.1% (n = 3 assays), and the intra-assay covariance for extraction and assay was 13.7% for a neonatal bovine plasma QC sample containing 94.2 ng/mL of IGF2.

### 2.9 Expression of genes from the IGF system in placental and fetal tissues

Relative expression levels of *IGF1*, *IGF2*, *IGF1R*, *IGF2R*, *INSRA*, *INSRB*, and *IGFBP1-6* were determined by real-time quantitative PCR (qPCR). RNA was extracted from the brain (telencephalon), heart (apex), liver (*Lobus hepatis sinister*), skeletal muscle (*M. semitendinosus*), and placenta (cotyledon, obtained from the second largest placentome close to the fetus) tissue using TRI Reagent (Ambion, Thermo Fisher Scientific, Melbourne, Australia) in combination with ceramic beads (MoBio Laboratories, Carlsbad, CA, USA) and a Precellys 24 tissue homogenizer (Bertin Technologies, Montigny-le-Bretonneux, France) following the manufacturer’s instructions. RNA quantity and quality were assessed by a NanoDrop ND-1000 spectrophotometer (Thermo Fisher Scientific, Melbourne, Australia) and Agilent RNA 6000 Nano Kit with Bioanalyzer 2100 (Agilent Technology, Santa Clara, CA). RNA integrity number averaged 8.4 for the brain, 8.5 for the heart, 8.1 for the liver, 8.2 for the skeletal muscle, and 7.2 for the placenta. After DNase treatment (RQ1-DNase, Promega, Madison, WI, USA), 500 ng RNA was reverse transcribed using Superscript III First-Strand cDNA synthesis kit (Invitrogen, Thermo Fisher Scientific, Melbourne, Australia) with random hexamer oligonucleotides following the manufacturer’s instructions. Real-time qPCR reactions were performed in an Eppendorf Mastercycler ep realplex (Eppendorf Inc. Hamburg, Germany) using 4 µL of 40-fold or 5.2 µL of 20-fold diluted cDNA, 0.8 µL of 5 pmol/µL primer mix, and 6 µL of FastStart Universal SYBR Green Master (Roche Diagnostics, North Ryde, Australia) in a total volume of 12 µL. A standard curve comprised of a twofold serial dilution of pooled cDNA template for each fetal tissue over eight data points, and a non-template control was included in each qPCR experiment. Fetal cDNA samples were measured in duplicate and cDNA standard curve data points in triplicate. Primer sequences and other details of amplicons from 12 target and five reference housekeeping genes are shown in **Supplementary Table S2**. Target gene transcript abundance was normalized to the geometric mean of the transcript abundances of the most stably expressed reference genes selected for each tissue using NormFinder (41).

### 2.10 Statistical analysis

We used the general linear model procedure of SAS (SAS University, SAS Inst.) and performed ANOVA to determine the effects of fetal sex adjusted for fetal genetic effects on investigated parameters using the model:


$${\text{y}}_{\text{ij}}={\text{S}}_{\text{i}}+{\text{G}}_{\text{j}}+{\text{e}}_{\text{ij}}$$

where *y_ij_* were conceptus parameters, *S* (*_i_* = male, female) was the fetal sex effect, and *G* (*_j_* = A×A, B×A, A×B, and B×B) was the fetal genetic effect.

Least square means with standard errors of means for male and female fetuses were computed for parameters with overall model significance of *p*<0.05 and compared using two-tailed *t*-test with a significance threshold of *p*<0.05. Data with residuals that failed to follow normal distribution and could not be normalized by logarithmic transformation were tested by Wilcoxon two-sample test with a significance threshold of two-sided *p*< 0.05.

To determine sex-specific relationships between gross morphological, histomorphological, endocrine, and clinico-chemical parameters, the independent variable of interest was nested within fetal sex and sex-specific regression slopes were derived using the model:


$${\text{y}}_{\text{i}}={\text{S}}_{\text{i}}+\text{X }\left({\text{S}}_{\text{i}}\right)+{\text{e}}_{\text{i}}$$

where *y_i_* was the response variable, *S* (*_i_* = male, female) was the intercept estimate for fetal sex, and *X*(*S_i_*) corresponded to the independent variable nested within fetal sex. Sex-specific regression slopes and Pearson correlation coefficients were considered significant at *p*<0.05. Regressions and correlations were not adjusted for genetics in order to be able to show regression slopes and correlation coefficients with actual data points.

## 3 Results

### 3.1 Asymmetric lower growth of the female fetus

**Figure 1:**
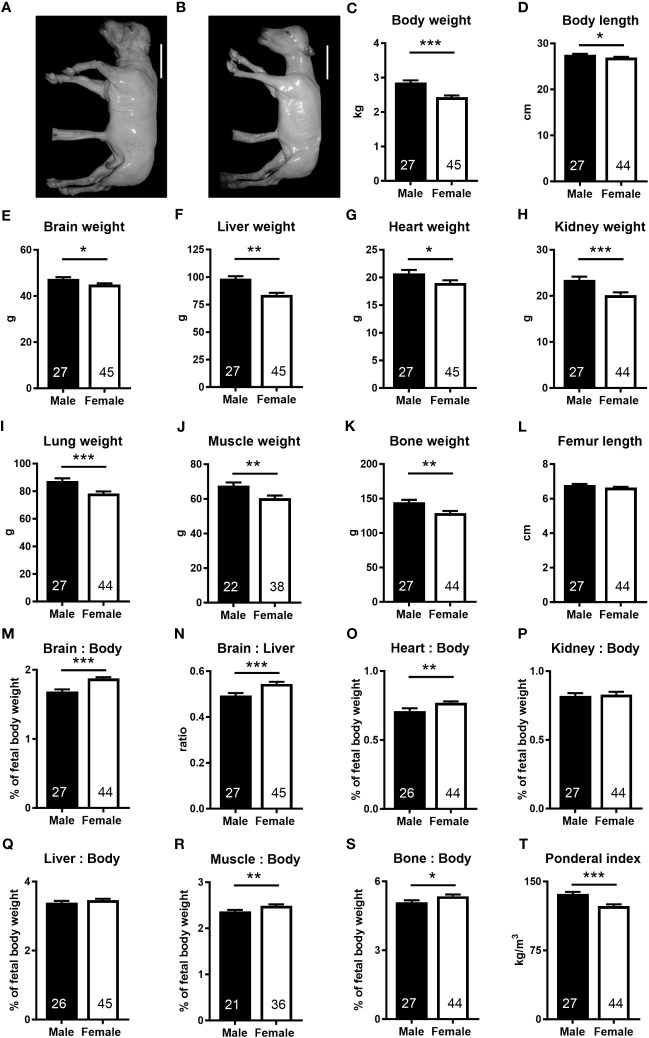
The female fetus displays asymmetric lower growth at mid-gestation. **(A, B)** Representative photographs of male and female fetuses recovered at mid-gestation (Day 153 ± 1, 55% term). Scale bars: 10 cm. **(C–I)** Wet weights obtained after removal of fetuses from uteri. **(D)** Body length, measured as spinal column length, including cervical, thoracic, and lumbar vertebrae. **(J, K)** Muscle and bone mass based on dissected muscles and bones. **(M, N)** Brain to body and brain to liver weight ratios, **(O)** heart to body weight ratio, **(P)** kidney to body weight, and **(Q)** liver to body weight ratio. **(R, S)** Relative muscle and bone mass and **(T)** ponderal index. Data are presented as least square means ± SEM with numbers of individuals indicated inside bars. **(C–E, G–T)** Two tailed *t*-test was used. *p<0.05, **p<0.01, ***p<0.001. **(F)** Wilcoxon two-sample test was used. Ws=1,242, z=2.97, **p<0.01.

We took advantage of the diverse genetics available in the outbred bovine to generate under standardized conditions a resource of healthy concepti from uncomplicated pregnancies at mid-gestation (week 21, 55% term) with fetal weights ranging from 1.75 to 3.98 kg (**Supplementary Table S1**). Female fetuses weighed less (−15.0%), had lower skeletal muscle (−10.7%) and bone mass (−10.8%), and were shorter (−3.6%) and thinner (ponderal index, −9.6%) than male fetuses. Absolute weights of the brain (−5.2%), liver (−14.9%), heart (−8.4%), kidney (−14.1%), and lung (−10.5%) were lower in female fetuses. However, relative to fetal weight, brain (+11.3%), heart (+8.5%), skeletal muscle (+4.6%), and bone (+5.3%) weights were higher in female than in males. The brain to liver weight ratio was also higher in females (+10.3%) than in males (**Figure 1**).

### 3.2 Umbilico-placental supply implicated in asymmetric lower female growth

#### 3.2.1 Sex differences in umbilico-placental phenotype

**Figure 2:**
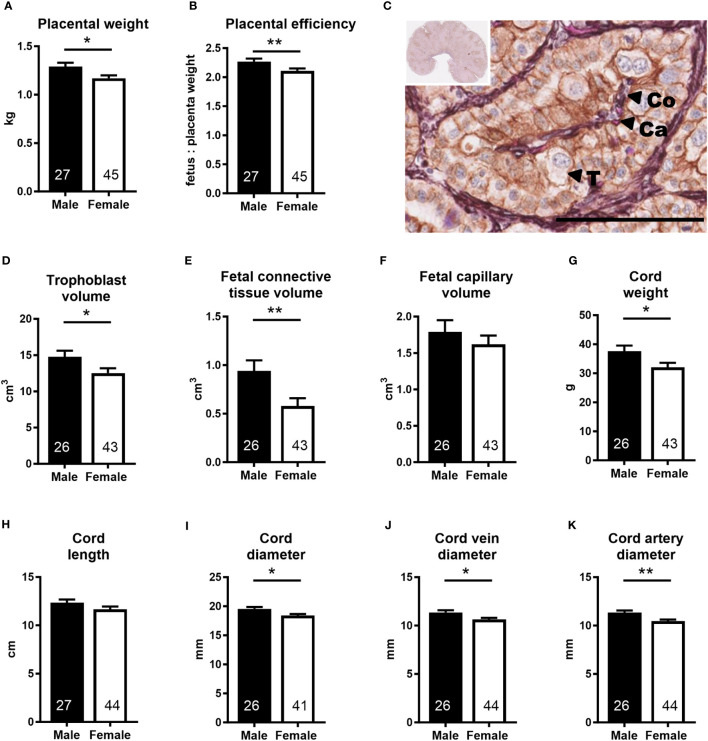
Placenta and umbilical cord parameters indicate lower umbilico-placental supply in the female conceptus. **(A)** Placental weight and **(B)** placental efficiency at mid-gestation (Day 153 ± 1, 55% term). **(C)** Representative photomicrograph shows histological detail of the placenta (insert). Scale bar is 100 µM. Double label immunohistochemistry with hematoxylin and eosin as counterstains differentiates placental tissue components. T, trophoblast; Co, fetal connective tissue; Ca, fetal capillary. **(D–F)** Placental tissue volumes calculated as volume density multiplied by the weight of the sample. **(G–K)** Umbilical cord characteristics. Data are least square means ± SEM with numbers of individuals indicated inside bars. **(A, B, D, E, G, H, J, K)** Two-tailed *t*-test was used. *p<0.05, **p<0.01. **(F, I)** Wilcoxon two-sample test was used for fetal capillary volume, Ws=958, z=0.58, *p*>0.05 and for cord diameter, Ws=1,043, z=2.04, *p<0.05.

Placental (−9.3%) and umbilical cord (−14.7%) weights, placental efficiency (−7.1%), umbilical cord diameter (−6.1%), and umbilical vein (−6.5%) and artery (−7.8%) diameters were lower in females than in males, while umbilical cord length was similar in both sexes (**Figure 2**). In the female placenta, trophoblast volume (−15.2%), fetal connective tissue volume (−38.3%), and fetal connective tissue volume density (−32.0%) were also lower than in male placentas (**Figure 2**; **Supplementary Table S3**). All other placental tissue components and measurements of maternal and fetal exchange surface were similar in both sexes (**Supplementary Table S3**).

#### 3.2.2 Sex differences in clinico-chemical parameters and the IGF system

**Figure 3:**
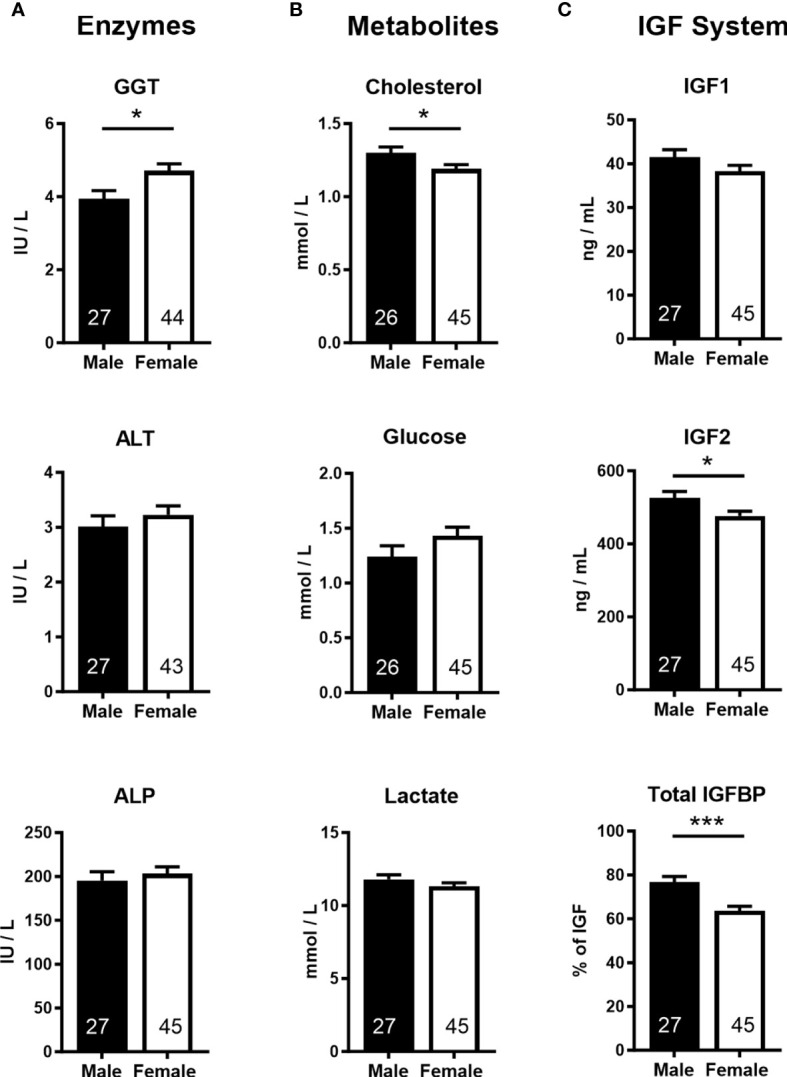
Cord blood parameters implicate altered nutrient supply and liver growth in asymmetric lower growth of the female conceptus at mid-gestation (Day 153 ± 1, 55% term). **(A)** Enzymes; γ-glutamyl transferase (GGT), alanine transaminase (ALT), alkaline phosphatase (ALP). **(B)** Metabolites; cholesterol, glucose, lactate. **(C)** Insulin-like growth factor (IGF) system; insulin-like growth factor 1 and 2 (IGF1, IGF2), combined insulin-like growth factor binding proteins (IGFBP). Data are least square means ± SEM with numbers of individuals indicated inside bars. With the exception of IGF1, a two-tailed *t*-test was used to calculate statistical difference. *p<0.05, ***p<0.001. Wilcoxon two-sample test for IGF1: Ws=1,029, z=0.5, *p*>0.05.

**Figure 4:**
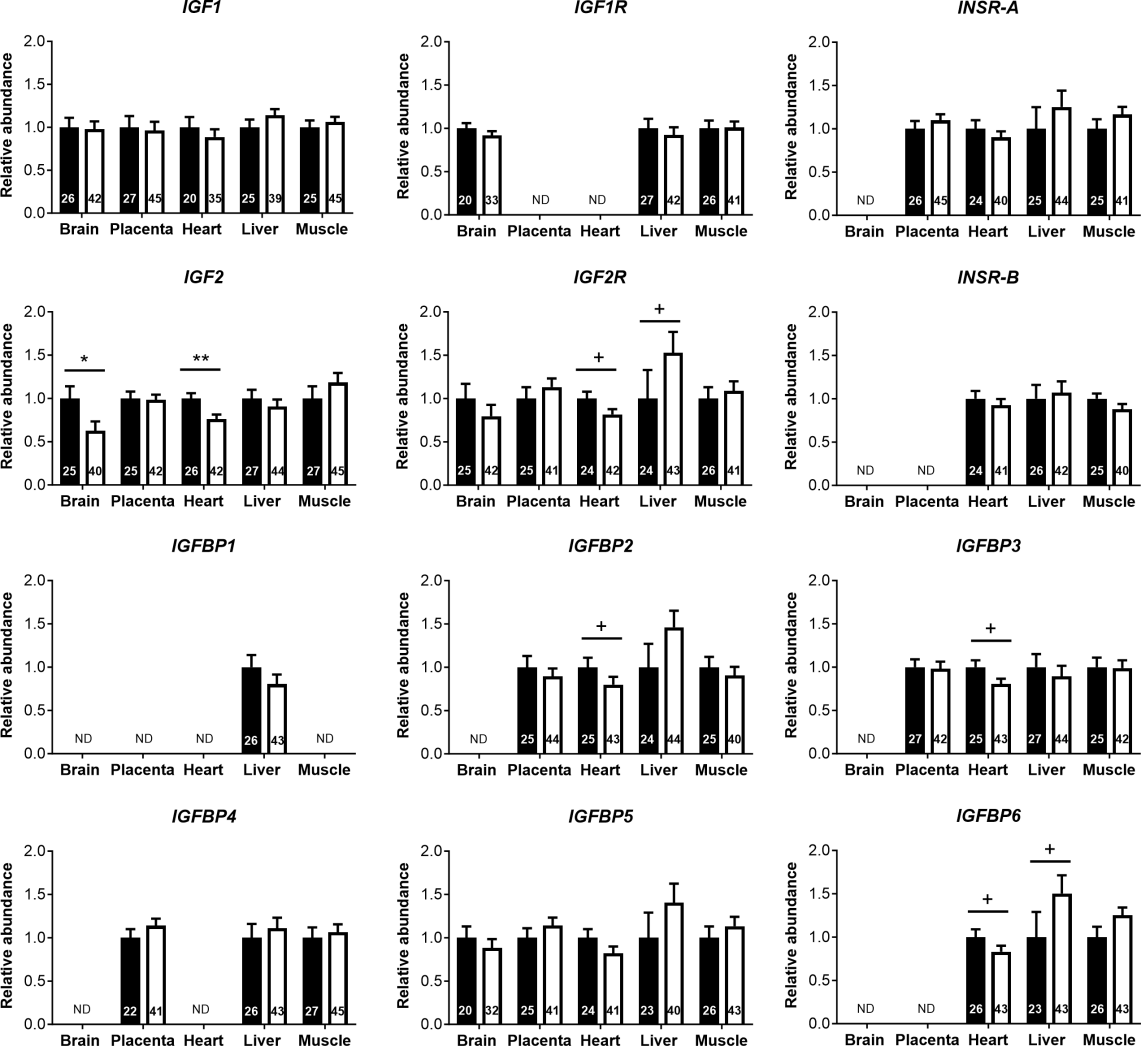
Transcript abundances for genes of the insulin-like growth factor (IGF) system in tissues of male and female concepti implicate IGF2 in asymmetric lower growth of the female conceptus at mid-gestation (Day 153 ± 1, 55% term). Transcript abundances measured for females (white bar) are compared within the tissue relative to values obtained for males (black bar). Data are least square means ± SEM relative to males set as 1 with numbers of individuals indicated inside bars. PCR primers and annealing temperatures for target and reference housekeeping genes are presented in **Supplementary Table S2**. ND, transcript not detected. Two-tailed *t*-test was used to calculate statistical difference between groups. ^+^ *p*<0.10; *p<0.05; **p<0.01. Wilcoxon two-sample test was used for *IGFBP2* in heart, ^+^ *p*<0.10.

Analyses of cord blood serum revealed that γ-glutamyl transferase activity and cholesterol concentration were higher (+19.5%) and lower (−8.5%), respectively, in females than in males (**Figures 3A, B** **),** while 16 other parameters, including glucose and lactate concentrations, were similar in both sexes (**Supplementary Table S4**). Circulating plasma IGF2 (−9.6%) and total IGFBPs (−17.0%), but not IGF1, were lower in females than in males (**Figure 3C**). Furthermore, measurement of transcripts from 12 genes of the IGF system in five fetal tissues indicated lower abundance of *IGF2* transcript in the female brain (−37.5%) and heart (−23.7%), but *IGF2* expression in the liver, skeletal muscle, and placenta was similar for both sexes (**Figure 4**).

### 3.3 Sex-specific umbilico-placental supply is reflected in phenotypic relationships between conceptus characteristics

**Figure 5:**
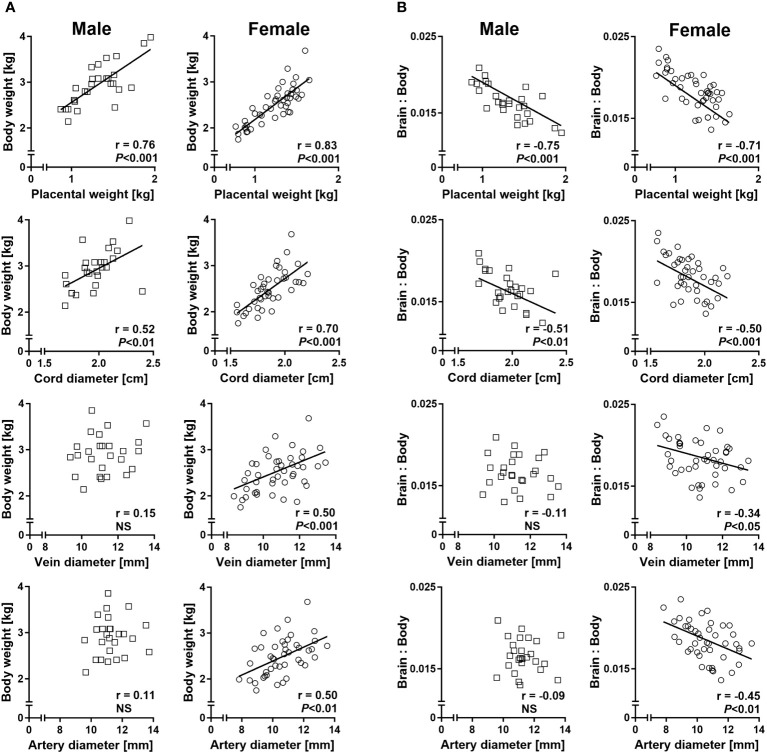
Sex-specific phenotypic relationships of conceptus characteristics reflect sex-specific umbilico-placental supply at mid-gestation (Day 153 ± 1, 55% term). **(A)** Relationships of fetal body weight with placental weight and umbilical cord characteristics. **(B)** Relationships of brain to body weight ratio with placental weight and umbilical cord characteristics. Highly similar relationships were obtained for brain to liver weight ratio and are presented in **Supplementary Figure S2**. Regression lines for significant relationships and Pearson product moment correlation coefficients with *p*-values are indicated. NS, not significant; *p*>0.05.

Sex-specific regression analyses of fetal body weight on placental weight and umbilical cord diameter revealed strong positive relationships in both males and females. However, significant positive relationships between fetal body weight and umbilical vein and artery diameters were only present in females (**Figure 5A**). Similarly, relationships of liver weight with umbilical vein and artery diameters were female specific (**Supplementary Figure S1**). Regressions of the brain to body weight ratio on placental weight and umbilical cord diameter indicated strong negative relationships in both sexes, but only females displayed significant negative relationships of brain to body weight ratio with umbilical vein and artery diameters (**Figure 5B**). Female-specific relationships were also observed between brain to liver weight ratio and umbilical vessel diameters (**Supplementary Figure S2**).

**Figure 6:**
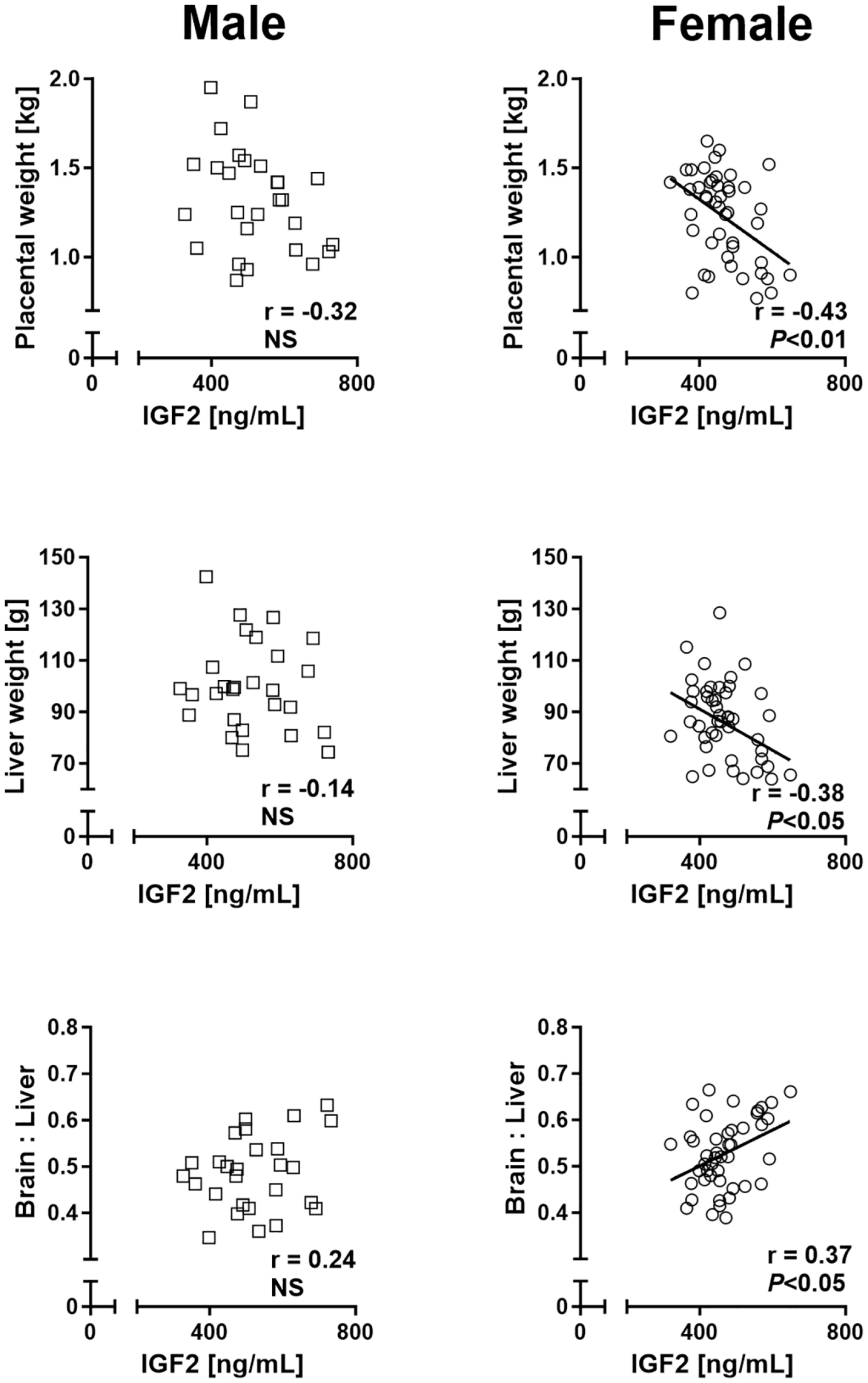
Phenotypic relationships provide evidence for sex differences in involvement of the insulin-like growth factor axis in nutrient demand signaling at mid-gestation (Day 153 ± 1, 55% term). Relationships of placental and fetal liver weights and brain to liver weight ratio with circulating fetal insulin-like growth factor 2 (IGF2) in male (left panel) and female (right panel) concepti. Regression lines for significant relationships and Pearson product moment correlation coefficients with *p*-values are indicated. NS, not significant; *p*>0.05.

Regression analyses of placental and liver weights on cord plasma IGF2 concentrations revealed significant negative relationships for female but not male concepti, while fetal weight was not associated with circulating IGF2. Moreover, brain to liver weight ratio displayed a female-specific positive relationship with circulating IGF2 (**Figure 6**).

## 4 Discussion

We combined prenatal phenotypic, histomorphological, clinico-chemical, endocrinological, and gene expression analyses in a single large animal species to investigate sex-specific differences in fetal growth strategies. At mid-gestation, upon entering accelerated growth, females had a smaller placenta with lower body and organ weights, but higher brain to body, brain to liver, and heart to body weight ratios than males. Females were also shorter and thinner and had a higher relative muscle and bone mass. To further assess the contribution of the placenta to sex-specific phenotypic asymmetry, we characterized morphology and histomorphology of the placenta and umbilical cord and their relationships with fetal biometry. Significant female-specific relationships of brain to body and brain to liver weight ratios with cord vessel diameters suggest that smaller size of critical umbilico-placental structures creates a growth-limiting environment for the healthy female conceptus. Higher γ-glutamyl transferase activity and lower cholesterol concentration in the cord serum of females may reflect reduced growth and/or maturity of the female fetal liver (42–44), associated with altered umbilico-placental hemodynamics. The lower insulin-like growth factor 2 (*IGF2*) expression in the brain and heart of the female fetus and lower circulating IGF2 implicate female-specific modulation of these key endocrine mediators of growth by nutrient supply. Taken together, these findings indicate that, by mid-gestation, the female conceptus is in an asymmetric slower growth mode compared to males.

Lower placental weight and efficiency in females in the present study are consistent with previous data (15, 19, 45), suggesting sex-specific substrate supply to the fetus. However, our histomorphometric analyses of the placenta revealed additional differences between sexes, including lower trophoblast and fetal connective tissue volumes in females. Trophoblast cells in close apposition to the maternal epithelium facilitate nutrient transfer (46) and fetal placental connective tissue contains myofibroblasts whose contractile properties can facilitate movement of substrates across the placental barrier (47, 48). Together, our data therefore suggest a mechanism where differences in placental structure and function contribute to lower nutrient transfer to the female fetus. Substrates from trophoblast cells enter the fetal placental capillary networks that culminate in the umbilical cord vessels (49). In the present study, placental capillary volume and volume density did not differ between sexes, but umbilical cord vein and artery diameters of female concepti were significantly smaller than those of males. In the course of uncomplicated pregnancy, changes in umbilical vessel diameter correlate with changes in blood flow (50, 51), and in pregnancies with fetal growth restriction, lower umbilical cord vein cross-sectional area is associated with reduced venous flow (52). The similar length of the umbilical cord in male and female fetuses in our study may exacerbate the effects of smaller female umbilical vessel diameters on blood flow and nutrient supply, as blood flow resistance is proportionate to umbilical vessel length (53). Furthermore, thicker cords contain higher volumes of Wharton’s jelly, which provides a protective layer for umbilical cord vessels and may influence blood flow to the fetus (54); lower cord diameter of females in the present study may reflect less Wharton’s jelly (55). In any case, cord compression selectively increases resistance, especially in the venous outflow tract (53, 56), and resistance to blood flow in smaller umbilical cord vessels of females is thus amplified by their thinner cord. In human, female fetuses had higher umbilical artery pulsatility index than male fetuses during gestation weeks 20–36 (57) and a tendency towards lower umbilical blood flow per kilogram estimated fetal weight at 22–24 weeks (58), a similar stage of gestation as fetuses from the present study. Overall, our findings of an umbilical cord phenotype that is thin, with smaller vein and artery diameters in females, suggest that lower umbilical blood flow may contribute to asymmetric reduced fetal growth in the female at mid-gestation and that further studies to assess these potential sex-specific differences in umbilical blood flow are warranted.

The relationships between fetal and placental and umbilical cord parameters reported here provide further evidence for sex-specific differences in blood supply to the fetus. In both sexes, we observed strong positive relationships of fetal body and liver weight with placental weight and umbilical cord diameter, but only females also displayed similar positive relationships of fetal body and liver weight with cord vessel diameters. These relationships suggest that placental weight and umbilical cord diameter are general determinants of fetal growth, while cord vessel diameter has critical growth-regulating effects in the female fetus only. In the small-for-gestational-age human fetus, brain to liver volume ratio is negatively correlated with umbilical venous volume flow relative to fetal weight (59), consistent with a restriction of fetal blood flows by smaller umbilical cord vein and artery diameters. Preferential blood flow redistribution to the brain and heart at the expense of other organs, such as the liver, provides a compensatory adaptive mechanism in the intrauterine growth restricted human fetus and in animal models of restricted placental substrate supply (60–62). Negative relationships of fetal brain to liver and fetal brain to body weight ratios with umbilical vein and artery diameters in females, but not males, suggest that sex-specific phenotypes of both umbilical vein and artery contribute to asymmetric growth with brain “sparing” observed in females in the present study.

High levels of cord blood γ-glutamyl transferase (GGT) activity at birth have previously been linked to hepatic immaturity (42) and placental insufficiency (43). The higher GGT activity in the cord blood of the female conceptus together with lower female placental capacity reported here is therefore consistent with a less mature female liver and lower placental supply. Furthermore, we found that only in females were cord vessel diameters positive predictors of absolute liver weight, an indication of altered blood flow to the liver, which may limit growth and maturation. Finally, female cholesterol levels were lower than male cholesterol levels. Fetal *de novo* cholesterol synthesis occurs predominantly in the liver, and demand for cholesterol is positively related to fetal growth rate (44). Thus, it appears that serum cholesterol is also affected by altered hemodynamics and lower perfusion and growth of the female fetal liver.

The insulin-like growth factor (IGF) system is a major regulator of conceptus growth (24), and fetal circulating IGF1 increases throughout gestation, while IGF2 increases as gestation progresses and then declines in the final third of gestation (40, 63). Consistent with the described dominant role of IGF2 in early fetal growth and development (24), we found that cord blood IGF2, but not IGF1, levels were lower in the female than male conceptus at mid-gestation. In addition, IGF2 gene expression in the brain and heart tissue of females was lower than in males, while IGF1 gene expression was similar for both sexes in all tissues studied. As fetal IGF expression is responsive to changes in nutrition (24, 64), this finding points to female-specific restrictions in placental nutrient supply. Although we found no relationships between fetal weight and fetal circulating IGF2 in either sex, IGF2 in females was positively related to brain to liver weight ratio and negatively related to placental and liver weights. This supports the view that IGF2 may act as a fetal demand signal to the placenta, as previously demonstrated by gene deletion studies in mouse (65, 66) and is consistent with growth-promoting actions of IGF2 at mid-gestation through the placenta (24, 67, 68). Growth-promoting effects of IGFs are regulated by binding proteins (IGFBPs), which transport IGFs in plasma, increase their half-life, and regulate the availability of free IGFs (69). We found lower cord serum total IGFBP levels in females at mid-gestation, potentially a compensatory mechanism to increase IGF availability in response to lower circulating IGF2. The mechanisms for differences in circulating IGFBPs are unclear; we found no sex differences in the expression of IGFBP1-6 genes in the fetal brain, heart, liver, skeletal muscle, or placenta. However, lower circulating IGF2 levels observed in females were accompanied by similar differences in IGF2 gene expression in fetal heart (see above), an organ that also displayed consistent trends (each *p*<0.10) for lower expression of IGFBP2, IGFBP3, and IGFBP6 genes in females. Thus, the present data indicate sex-specific expression of multiple components of the IGF axis.

The sex-specific differences observed in the present study at different experimental levels may be explained by an evolutionary biology perspective on developmental programming. The parallels between, and the complementarity of, the Trivers–Willard (70) and Developmental Origins of Health and Disease (DOHaD) hypotheses were pointed out and explored by Aiken and Ozanne, 2013 (6). In this context, the Trivers–Willard hypothesis, which states that in a species where there is a sex-based difference in reproductive success, mothers with plentiful resources will be able to invest in the sex with a reproductive disadvantage, whereas mothers facing an adverse environment will preferentially produce offspring of the sex with a greater chance of reproductive success, can explain sex-specific results of developmental programming, if developmental programming is considered as the molecular mechanism by which this differential investment in offspring sex can be realized (6). The female-specific adaptations revealed in our study thus provide a basis for increased genetic fitness of female concepti under adverse environmental conditions as postulated (6). As sex-specific differences in mammalian growth and development emerge early, from the pre-implantation embryo stage onward, before the onset of hormone production in the developing gonads of the embryo (71, 72), they are most likely caused by genetic differences arising from female XX and male XY chromosome complements (73). We propose gametologues, a distinct class of evolutionary conserved X–Y chromosome paired genes that are located outside the recombining pseudo-autosomal region of the sex chromosomes, as potential drivers of sex-specific phenotypic differences in the context of developmental plasticity. Gametologues are part of the epigenetic machinery and/or global regulators of gene activity with functions in cell cycle regulation, cell proliferation, and growth control, including brain development and angiogenesis (74). These genes are thus prime candidates for further studies on sex-specific developmental programming.

We have demonstrated that the healthy female conceptus at mid-gestation is in an asymmetric growth-limiting mode of development. Female-specific relationships of fetal body weight and brain to liver weight ratios with umbilical cord artery and vein diameter indicate that the umbilico-placental vascular system is growth-limiting for female fetuses. The reduction in size of critical umbilico-placental structures thus creates a scenario where the female conceptus reorganizes distribution of blood flow to favor brain and heart growth. Higher GGT and lower cholesterol levels in females as markers of reduced liver perfusion, are consistent with this phenotype, while lower circulating IGF2 suggests restricted nutrient supply or lower nutrient demand. This phenotype is not transient, as higher brain to body weight and brain to liver weight ratios of females are also evident in bovine fetuses at term (75). We conclude that the female prenatal growth strategy with adaptation to lower umbilico-placental substrate supply may (i) have long lasting and fundamental physiological effects *per se*, (ii) confer resilience to growth-restricting changes in the intrauterine environment later in pregnancy (22, 76) with lower risk of adverse perinatal outcomes (77, 78), and (iii) contribute to sex bias in programming, including susceptibility to non-communicable diseases.

Our study affirms the need for fetal sex-specific prenatal care to minimize unfavorable health outcomes. In combination with non-invasive technologies for morphological and functional assessment of concepti, our findings may contribute to the development of sex-specific thresholds and interventions at crucial time points for prenatal growth and development to identify and treat fetuses at risk of suboptimal development.

## Data availability statement

The raw data supporting the conclusions of this article will be made available by the authors without undue reservation. Original gene expression data presented in the study are publicly available. This data can be found here: https://doi.org/10.25909/24901491.v1.

## Ethics statement

The animal study was approved by The University of Adelaide, Animal Ethics Committee. The study was conducted in accordance with the local legislation and institutional requirements.

## Author contributions

CE: Data curation, Formal analysis, Investigation, Methodology, Validation, Visualization, Writing – original draft, Writing – review & editing. KG: Data curation, Investigation, Methodology, Resources, Validation, Writing – review & editing. RX: Formal analysis, Investigation, Writing – review & editing. AJ: Investigation, Writing – review & editing. MG-S: Investigation, Writing – review & editing. GN: Formal analysis, Methodology, Writing – review & editing. ES: Investigation, Writing – review & editing. MM: Resources, Writing – review & editing. IB: Investigation, Resources, Writing – review & editing. DT: Investigation, Project administration, Writing – review & editing. VC: Writing – review & editing. JO: Methodology, Resources, Writing – review & editing. CR: Methodology, Resources, Supervision, Validation, Writing – review & editing. SH: Supervision, Validation, Visualization, Writing – original draft, Writing – review & editing, Conceptualization, Data curation, Formal analysis, Funding acquisition, Investigation, Methodology, Project administration, Resources. KK: Formal analysis, Investigation, Methodology, Project administration, Supervision, Validation, Writing – original draft, Writing – review & editing.
