# Supplementary material for: Asymmetric growth-limiting development of the female conceptus
Source: Front Endocrinol (Lausanne). 2024 Feb 1;14:1306513. doi: 10.3389/fendo.2023.1306513 (PMC10867182; doi:10.3389/fendo.2023.1306513)
Supplement: Supplementary file 1 [file DataSheet_1.pdf]

Supplementary Material

Asymmetric growth-limiting development of the female conceptus

Consuelo Amor S. Estrella[1,2‡], Kathryn L. Gatford[1,3], Ruidong Xiang[1,2], Ali Javadmanesh[1,2], Mani Ghanipoor-Samami[1,2], Greg S. Nattrass[4], Entesar Shuaib[1,2], Milton M. McAllister[5], Ian Beckman[5], Dana A. Thomsen[1,2], Vicki L. Clifton[1, 6], Julie A. Owens[1, 7], Claire T. Roberts[1,8], Stefan Hiendleder[1,2†*], Karen L. Kind[1,2†*]

*** Correspondence:**
Corresponding Authors: Karen Kind and Stefan Hiendleder
karen.kind@adelaide.edu.au; stefan.hiendleder@adelaide.edu.au

1 Supplementary Figures and Tables

1.1 Supplementary Figures

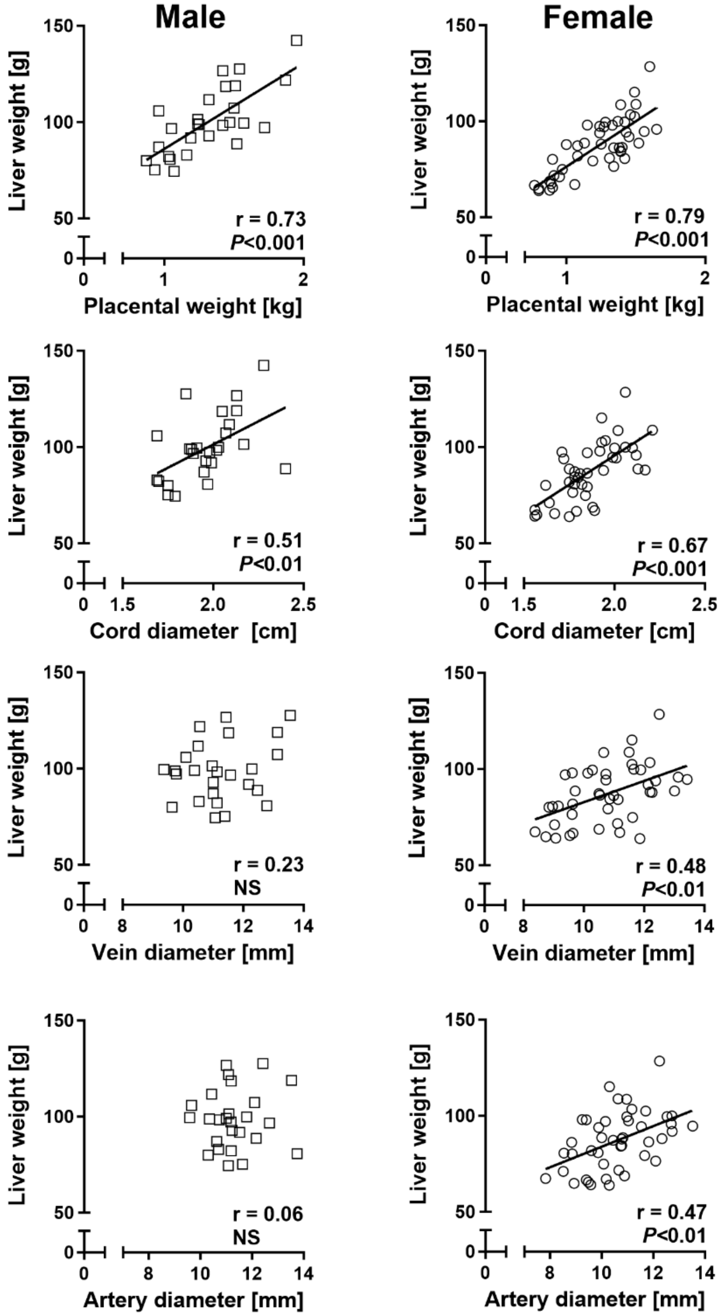

Supplementary Figure 1. Relationships of liver weight with placental weight and umbilical cord characteristics in male and female concepti at mid-gestation (Day 153±1, 55% term). Vein: Umbilical cord vein. Artery: Umbilical cord artery. Regression lines for significant relationships, Pearson product moment correlation coefficients and *P*-values are indicated. NS: not significant, *P*>0.05.

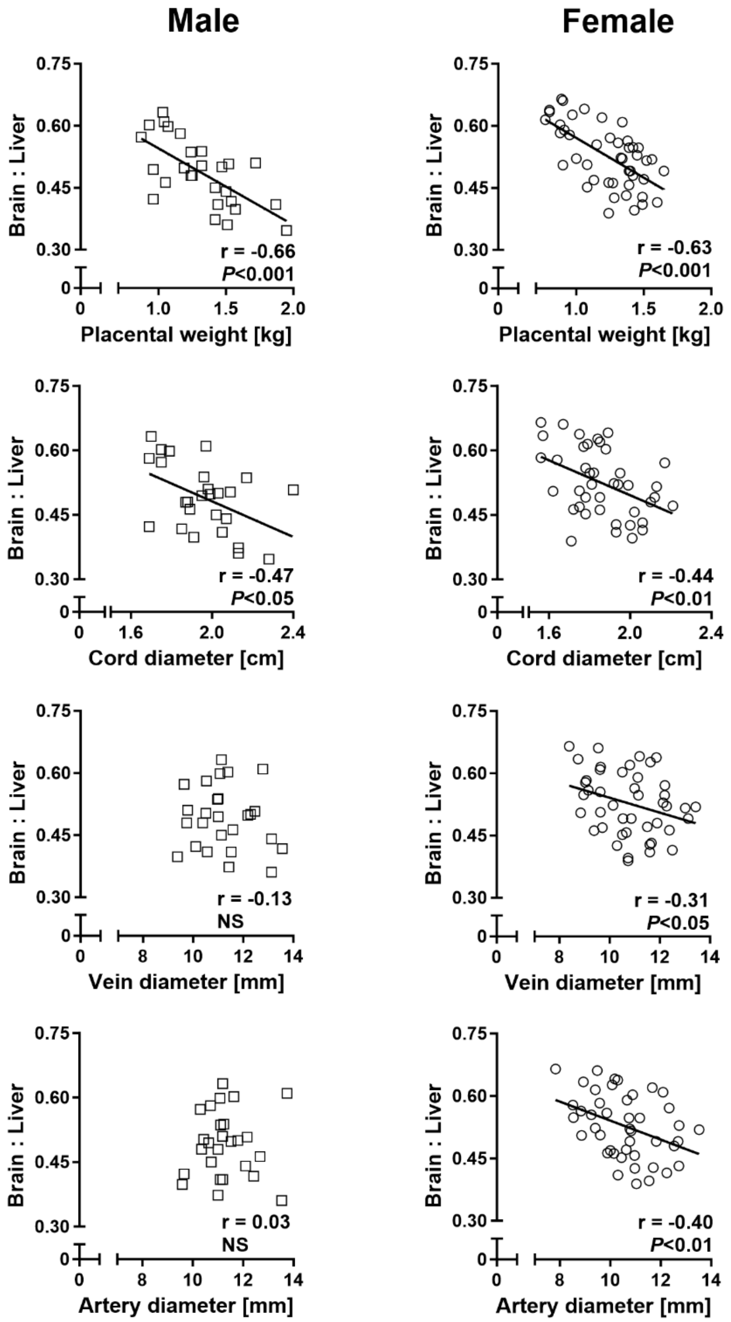

Supplementary Figure 2. Relationships of brain to liver weight ratio with placental weight and umbilical cord characteristics in male and female concepti at mid-gestation (Day 153±1, 55% term). Vein: Umbilical cord vein. Artery: Umbilical cord artery. Regression lines for significant relationships, Pearson product moment correlation coefficients and *P*-values are indicated. NS: not significant, *P*>0.05.

1.2 Supplementary Tables

Table S1. Numbers and weights for male (M) and female (F) fetuses with defined *Bos taurus taurus* (A) and *Bos taurus indicus* (B) genetics at mid-gestation (Day 153±1, 55% term). Sire genetics listed first. Least square means ± SEM are shown. Individual fetal weights ranged from 1.75 to 3.98 kg.

**Fetal genetics**	**Sex**	**n**	**Weight (kg)**
AA	M	11	3.22 ± 0.10
AA	F	12	2.79 ± 0.10
AB	M	7	2.69 ± 0.12
AB	F	6	2.26 ± 0.13
BA	M	5	3.15 ± 0.14
BA	F	17	2.64 ± 0.08
BB	M	4	2.39 ± 0.16
BB	F	10	2.04 ± 0.10

Table S2. PCR Primers, annealing temperatures and size of fragments used in real-time quantitative PCR measurement of transcript abundances for reference housekeeping and insulin-like growth factor (IGF) system target genes.

Gene		Primers[A]	Temp[B]	Size[C]	Accession no.[D]
VPS4A	F	GAAGACAGAAGGCTACTCGGGTG	60	106	NM_001046615.1
	R	ACAGACCTTTTTGAAGTGTGTTGCT			
ACTB	F	CTCTTCCAGCCTTCCTTCCT	62	245	NM_173979.3
	R	CCAATCCACACGGAGTACTTG			
RPS9	F	TAGGCGCAGACGGGCAAACA	60	136	NM_001101152.2
	R	CCCATACTCGCCGATCAGCTTCA			
GAPDH	F	GGGTCATCATCTCTGCACCT	60	264	NW_003103940.1
	R	CATAAGTCCCTCCACGATGC			
H3F3A	F	ACTGCTACAAAAGCCGCTC	60	231	XM_003586223.1
	R	ACTTGCCTCCTGCAAAGCAC			
IGF1	F	GATGCTCTCCAGTTCGTGTGC	58	140	NW_003103925.1
	R	TCCAGCCTCCTCAGATCACAG			
IGF1R	F	GATCCCGTGTTCTTCTACGTTC	58	100	XM_606794.3
	R	AAGCCTCCCACTATCAACAGAA			
INSR-A	F	TCCTCAAGGAGCTGGAGGAGT	59	89	AJ488553
	R	TTTCCTCGAAGGCCTGGGGAT			
INSR-B	F	TCCTCAAGGAGCTGGAGGAGT	59	110	AJ320235
	R	TAGCGTCCTCGGCAACAGG			
IGFBP1	F	ACCAGCCCAGAGAATGTGTC	59	119	NW_003103902.1
	R	CTGATGGCATTCCAGAGGAT			
IGFBP2	F	CACATCCCCAACTGTGACAA	58	114	NW_001494682.3
	R	GATCAGCTTCCCGGTGTTAG			
IGFBP3	F	CTACGAGTCTCAGAGCACAG	58	103	NT_181996.1
	R	GTGGTTCAGCGTGTCTTCC			
IGFBP4	F	ATGTGCCTGATGGAGAAAGG	57	106	NM_174557.3
	R	GCCATCCTGTGACTTCCTGT			
IGFBP5	F	CAAGCCAAGATCGAAAGAGACT	60	85	NM_001105327.1
	R	AAGATCTTGGGCGAGTAGGTCT			
IGFBP6	F	GGAGAGAATCCCAAGGAGAGTA	60	100	NM_001040495.1
	R	GAGTGGTAGAGGTCCCCGAGT			
IGF2	F	CTTCGCCTCGTGCTGCTATG	60	134	NM_174087.3
	R	GTCGGTTTATGCGGCTGGAT			
IGF2R	F	GATGGTAATGAGCAGGCTTACC	60	123	NM_174352.2
	R	ATCTCCTCCATCAGCCACTC			

[A] Forward (F) and reverse (R) primers are given. [B] Annealing temperature. [C] Amplicon size in basepairs. [D] GenBank accession number of DNA sequence used for primer design.

Table S3. Histomorphometric placental parameters for male and female concepti at mid-gestation (Day 153±1, 55% term). Least square means ± SEM are shown.

	**Male**	**Female**	***P*-value**[A]
**Volume density of placental tissues, V_d**			
Maternal epithelium	0.413 ± 0.009	0.420 ± 0.007	0.5248
Maternal capillaries	0.062 ± 0.004	0.065 ± 0.003	0.5357
Maternal connective tissue	0.065 ± 0.004	0.071 ± 0.003	0.2700
Trophoblast	0.374 ± 0.007	0.358 ± 0.006	0.1096
Fetal capillaries	0.044 ± 0.003	0.046 ± 0.002	0.6283
Fetal connective tissue	0.025 ± 0.003	0.017 ± 0.002	0.0079
Others (maternal septa)	0.016 ± 0.003	0.021 ± 0.003	0.1548
**Volume of placental tissues, cm^3**			
Maternal epithelium	16.40 ± 1.02	14.74 ± 0.80	0.2026
Maternal capillaries	2.41 ± 0.18	2.28 ± 0.14	0.5713
Maternal connective tissue	2.62 ± 0.20	2.45 ±.0.16	0.4885
Trophoblast	14.77 ± 0.85	12.52 ± 0.67	0.0417
Fetal capillaries	1.79 ± 0.16	1.62 ± 0.12	0.5564
Fetal connective tissue	0.94 ± 0.11	0.58 ± 0.08	0.0075
Others (maternal septa)	0.64 ± 0.14	0.75 ± 0.11	0.5853
**Maternal and fetal exchange surface**			
Trophoblast surface density, cm^2/g	244.27 ± 5.18	246.22 ± 4.06	0.7672
Maternal surface density, cm^2/g	255.97 ± 5.30	264.23 ± 4.14	0.2227
Trophoblast surface area, cm^2	9547.31 ± 540.86	8634.57 ± 424.09	0.1893
Maternal surface area, cm^2	10117.42 ± 597.48	9292.49 ± 468.49	0.2818
Trophoblast barrier thickness, µM	15.40 ± 0.38	14.67 ± 0.30	0.1405
Maternal barrier thickness, µM	16.34 ± 0.43	16.02 ± 0.34	0.5665

[A] Two-tailed *t*-test was used to identify significant differences at $P<0.05$ except for fetal capillary volume ($P>0.05$) and volume densities of maternal septa ($P>0.05$) and fetal connective tissue ($P<0.01$), where Wilcoxon two-sample test was used.

Table S4. Cord serum clinico-chemical parameters for male and female concepti at mid-gestation (Day 153). Least square means ± SEM are shown.

	**Male**	**Female**	***P*-value[B]**
**Electrolytes, mmol/L**			
Total calcium	3.21±0.04	3.18±0.03	0.5494
Chloride	101.24±1.21	100.78±0.95	0.6035
Magnesium	1.37±0.03	1.37±0.02	0.8901
Phosphorus	2.46±0.06	2.40±0.04	0.4021
Potassium	7.55±0.18	7.19±0.14	0.1343
Sodium	137.15±1.49	135.96±1.17	0.5608
**Metabolites**			
Albumin, g/L	17.21±0.29	17.45±0.22	0.5223
Cholesterol, mmol/L	1.30±0.04	1.19±0.03	0.0156
Creatinine, umol/L	0.10±0.00	0.10±0.00	0.6244
Globulin, g/L	10.27±0.16	10.29±0.12	0.9482
Glucose, mmol/L	1.24±0.10	1.43±0.08	0.1341
Lactate, mmol/L	11.80±0.31	11.37±0.27	0.2314
Total protein, g/L	27.54±0.46	27.96±0.36	0.5364
Triglyceride, mmol/L	0.67±0.02	0.63±0.16	0.0870
Urea, mmol/L	5.14±0.40	4.54±0.32	0.2508
**Enzymes, IU/L[A]**			
ALP	195.33±10.14	203.04±7.96	0.5534
ALT	3.01±0.20	3.23±0.16	0.3215
GGT	3.94±0.23	4.71±0.19	0.0120
GLDH	4.81±1.36	7.35±1.08	0.1967

[A] ALP: Alkaline phosphatase; ALT: Alanine transaminase; GGT: γ-glutamyl transferase; GLDH: Glutamate dehydrogenase. [B] Two-tailed *t*-test was used to identify significant differences at $P<0.05$ except for chloride, sodium, creatinine and GLDH where Wilcoxon two-sample test was $P>0.05$.
